# Musculoskeletal problems among string instrumentalists in South Africa

**DOI:** 10.4102/sajp.v73i1.327

**Published:** 2017-02-03

**Authors:** Adedayo T. Ajidahun, Witness Mudzi, Hellen Myezwa, Wendy-Ann Wood

**Affiliations:** 1 Department of Physiotherapy, Faculty of Health Sciences, University of the Witwatersrand, South Africa

## Abstract

**Background:**

Musicians who play string instruments are affected more by musculoskeletal injuries when compared to other instrument playing groups. Musculoskeletal problems are commonly found in the upper extremities and trunk. Several risk factors such as gender, practice hours and instrument played are associated with the prevalence and distribution of musculoskeletal problems among string instrumentalists.

**Objectives:**

The aim of this study was to determine the prevalence, distribution, severity and risk factors for musculoskeletal problems among string instrumentalists.

**Method:**

A cross-sectional study design using both online and paper-based questionnaires were used to collect data from string instrumentalists playing in both amateur and professional orchestras in South Africa.

**Results:**

A total of 114 string instrumentalists participated in the study, of which 86 (77%) reported problems in one or more anatomic regions while 39 (35%) were currently experiencing musculoskeletal problems that affected their performance. The trunk and both shoulders were the most commonly affected body regions. The majority of the participants reported the severity of the complaints as mild to moderate with aching, soreness, tingling and fatigue being the most commonly used descriptors of the symptoms of playing-related musculoskeletal problems.

**Conclusion:**

The results of this study showed that the prevalence of musculoskeletal problems that affect performance is high among string instrumentalists in South Africa. An evaluation of associated risk factors with the aim of reducing injuries may be important in improving performance.

## Introduction

Musculoskeletal problems are common health problems of musicians, irrespective of the instrument played. Playing-related musculoskeletal disorder (PRMD) is an umbrella term that denotes musculoskeletal problems associated only with playing the musical instruments (Bragge, Bialocerkowski & Mcmeeken 2006). The most commonly reported symptoms of PRMDs are pain, tingling, loss of flexibility and weakness in the upper extremity and trunk (Brusky 2010; Paarup et al. 2011). The aetiology of PRMDs (a derivative of work-related musculoskeletal disorders) is multifactorial.

The occurrence of PRMDs is associated with several intrinsic factors such as joint hypermobility, age (Ranelli, Straker & Smith 2011; Yeung et al. 1999) and gender and extrinsic factors such as warm-ups, playing hours, playing position, posture and playing techniques (Kaufman-Cohen & Ratzon 2011). Psychosocial factors such as stage fright and anxiety also contribute to the development of musculoskeletal problems among musicians. This may be because of increased stress and resultant muscle tension (Williamon & Thompson 2006). The awkward posture normally assumed by string instrumentalists when playing is also considered as a contributing factor to the occurrence and location of PRMDs (Moraes & Antunes 2012).

Playing a string instrument increases the instrumentalists’ risk of musculoskeletal problems, mainly in the upper extremities but also in the trunk and sub-mandibular regions (Abréu-Ramos & Micheo 2007; Moraes & Antunes 2012). String instrumentalists have a higher risk of developing musculoskeletal problems when compared to other instrument groups (Hagberg, Thiringer & Brandström 2005). The occurrence of PRMDs is similar regardless of professional level; music students, amateurs and professionals all report similar prevalence of PRMDs (Bragge et al. 2006; Kok et al. 2013).

Lederman (2003) reported 69% prevalence of PRMDs among string instrumentalists. The distribution of musculoskeletal problems is specific to and closely related to the instrument. Neck, upper trapezius, right and left shoulders are the most common body areas of musculoskeletal problems among cellists (Rickert et al. 2012), whereas the neck, shoulders and the temporo-mandibular joint are the most commonly affected areas among violinists and violists (Moraes & Antunes 2012).

Female string instrumentalists have a higher prevalence of PRMDs when compared to their male counterparts (Davies & Mangion 2002). Also, older instrumentalists report musculoskeletal problems affecting performance more often than younger ones (Ranelli et al. 2011). Over the last three decades, studies have emanated from North America, Europe and Australia evaluating musculoskeletal problems of musicians. There is, however, still a dearth of literature on musculoskeletal injuries of musicians in Africa. In the last 5 years, only three small-sampled studies were found reporting the epidemiology of injury among musicians at various study sites within South Africa (Ajidahun & Phillips 2013; Barnes et al. 2011; Hohls 2010).

The studies were conducted in specific groups. Barnes et al. (2011) reported the injury profile of musicians in one professional orchestra. The injury profile of musicians was reported in two professional orchestras (Hohls 2010) and a school of music (Ajidahun & Phillips 2013). This study is a national survey of the prevalence of musculoskeletal injuries among string instrumentalists in South Africa.

The aim of this study was:

1. to establish the prevalence, distribution, severity and symptoms of PRMDs among string instrumentalists in South Africa
2. to determine the association between identified risk factors and the occurrence of PRMDs
3. to determine the relationship between the site of injury and the identified risk factors.

## Methods

### Study design

A cross-sectional study design using self-administered questionnaires was used to collect information about the prevalence, distribution and severity of musculoskeletal disorders of string instrumentalists in South Africa.

### Ethical consideration

This study was approved by the Human Research Ethics Committee (Medical) at the University of the Witwatersrand (M130836). Written informed consent was obtained from all the participants.

### Questionnaire

A 17-item self-administered questionnaire was modified from existing questionnaires in the literature (Berque, Gray & Mcfadyen 2014; Blackie, Stone & Tiernan 1999; Paarup et al. 2012). Questions relating to the prevalence, distribution or severity of musculoskeletal problems and symptoms of musculoskeletal injury were modified from literature (Berque et al. 2014; Blackie et al. 1999; Paarup et al. 2012). The questionnaire comprised three sections which included socio-demographics, practice experience and history of musculoskeletal injury. A pilot study was conducted to test the validity and reliability of the questionnaire. Expert opinion from professional musicians and music educators was sought to determine the validity of the modified questionnaire. The musicians and educators recommended the removal of one question related to pattern of play from the questionnaire. Music students at the University of the Witwatersrand were invited to complete the questionnaire for the reliability study. They completed the questionnaire twice with an interval of 24 hours (Paiva et al. 2014). Its test–retest reliability of the questionnaire showed moderate to strong reliability (*r*_p_ = 0.43–0.99).

The questionnaire was divided into three sections, which included socio-demographics, playing and practice experience, and history of musculoskeletal injury. A pilot study was conducted to test the reliability of the questionnaire. Expert opinion was used to determine the validity of the modified questionnaire.

### Sample and sampling

Bowed string instrumentalists (violinists, violists, cellists and double bassists) from both amateur and professional orchestras across the country were invited to participate in this study. Orchestras across the country were identified by using a snowball sampling technique. In order to identify the orchestras, a professional orchestra in Johannesburg was contacted and the manager of the orchestra gave information about the other orchestras. A total of 16 orchestras across 6 of the 9 provinces in South Africa were contacted to participate in this study. The eligibility criteria to participate required string instrumentalists to be aged 18 and above and not have any disabling neuro-musculoskeletal problems not associated with playing the musical instrument.

### Procedure

The self-administered questionnaire was administered in three ways – online survey, postage and on-site administration by the researcher. The online survey link was sent to the managers of the orchestras via email to forward to the string players in their organisation. In addition, some questionnaires were directly administered at the rehearsal venue of four orchestras and sent by post to another three orchestra companies. Follow-up was done via emails and phone calls regularly for a period of 1 year.

### Data analysis

Shapiro–Wilk test for normality rejected the null hypothesis (*p* < 0.05); therefore, non-parametric statistical tests were used to analyse the data. Central tendencies of median, range and frequencies were used to analyse the demographics, prevalence, distribution, symptoms and severity of the reported musculoskeletal problems. Association between dependent variables and independent variables were analysed using non-parametric tests: Fischer’s exact test and Mann–Whitney U tests as appropriate. Binary logistic regression was undertaken to analyse the relationship of variables that showed significant association with the dependent variables. Missing data were treated as missing in this study. All the statistical tests were conducted using the IBM SPSS 22.0 ®. Significance was set at *p* < 0.05.

## Results

### Response rate

Of the 16 orchestras contacted, only 6 agreed to send the online survey link to the string players in their orchestra, whereas others did not respond. Those that did not respond were further contacted via email, telephone or direct contact to complete the paper questionnaire. From this effort, a further seven orchestra agreed to participate and completed the questionnaires. Four of the orchestras completed the questionnaire on site and three received and returned the questionnaires through post. In order to ensure confidentiality, the respondents returned the questionnaire in a sealed envelope to the manager and all the questionnaires were posted together for cohesion and organisation purposes. The actual response rate for the online survey could not be determined because of non-disclosure of the number of string instrumentalists in the orchestra by some of the managers. To ascertain the number of string players in total, the number of string players was estimated based on those reported in large professional orchestras in South Africa from an earlier study (Hohls 2010). From this figure, the response rate for the online survey was 33.3%. A total of 52 respondents completed the online survey from 6 orchestras. The response rate of the questionnaires administered at the rehearsal venue was 100% (62/62) and by post was 15% (15/100). A total of 129 respondents participated in the study with an overall response rate of 40.9%, although only 114 respondents met the inclusion criteria. Table 1 outlines the response rate of the various data collection methods. The internal consistency of the modes of data collection was moderate for the online and paper-based questionnaires (α = 0.59–0.75).

**TABLE 1:** Response rate of participants.

Mode of survey	Number of orchestra	Mode of questionnaire return	Total number of questionnaires returned	Response rate (%)
Online	6	Survey Monkey^®^	52	33.3
Paper survey	7	Post/on site	15/62	15/100
**Total**	**13**	**-**	**129**	**40.9**

### Demographic information of the participants

The participants’ age ranged from 18 to 78 years with a median of 28 years. Of the 114 participants, 76 (67%) were women and 70 (62%) violinists while 5 (4%) were double bassists. The median of the years of experience and practice hours were 16 years and 9 hours per week. Table 2 presents the demographic information of the respondents.

**TABLE 2:** Demographic information and playing habits of participants.

Variables (*N* = 114)	Overall
**Age, median (range) years**	28 (60)
**Gender *n* (%)**	
Male	38(33)
Female	76(67)
**Instrument *n* (%)**	
Violin	70(62)
Viola	16(14)
Cello	22(20)
Double bass	5(4)
**Education *n* (%)**	
Postgraduate	32(29)
Bachelors	23(21)
Conservatory diploma	11(10)
Matriculation certificate	39(35)
Others	6(5)
**Practice hours per week, median (range)**	9 (45)
**Years of experience, median (range)**	16 (72)
**Dominant arm *n* (%)**	
Right	97(87)
Left	7(6)
Both	8(7)
**Playing position *n* (%)**	
Standing	28(25)
Low sitting	41(37)
High sitting	43(38)

**FIGURE 1:**
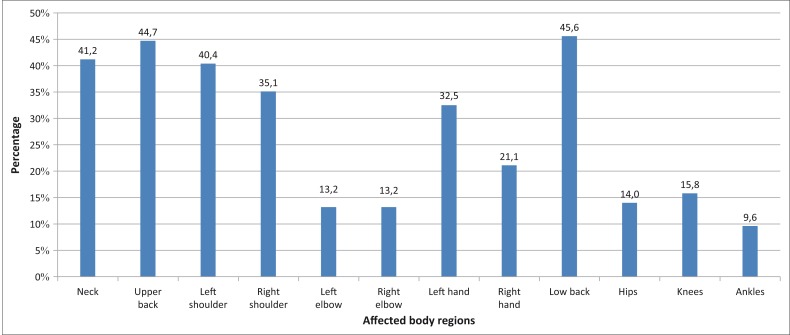
General distribution of playing-related musculoskeletal disorders in the 12 anatomic body regions among string instrumentalists.

The prevalence of musculoskeletal problems in one or more anatomic regions was 77% among the participants, with 35% experiencing problems that have interfered with their performance in the last 7 days. As illustrated in Figure 1, the areas of the low back (63%), upper back (54%), left shoulder (53%) and the neck (51%) were the most commonly affected body region, and lower extremities were the least reported areas of musculoskeletal problems. Figure 1 illustrates the general distribution of PRMDs among the respondents. The most common descriptors of the symptoms of PRMDs were aching (54%), soreness (41%), fatigue (41%) and tightening (39%).

**FIGURE 2:**
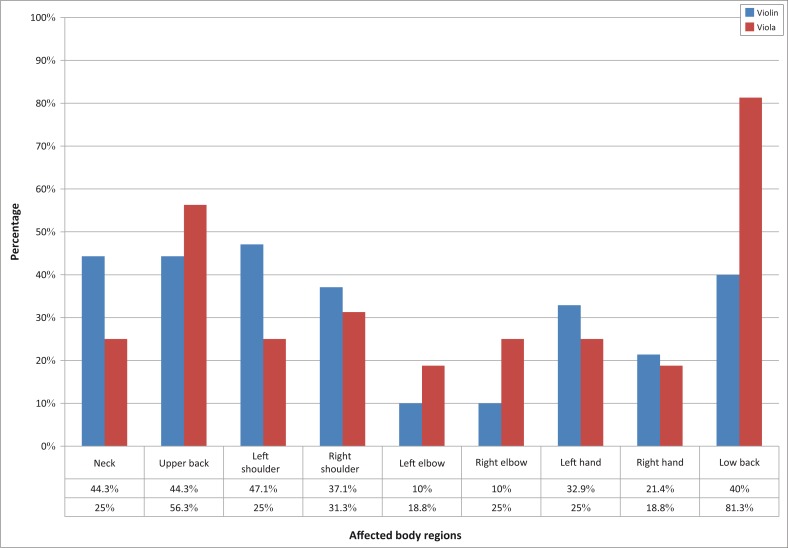
Distribution of musculoskeletal problems in the nine anatomic regions in upper string instrumentalists.

**FIGURE 3:**
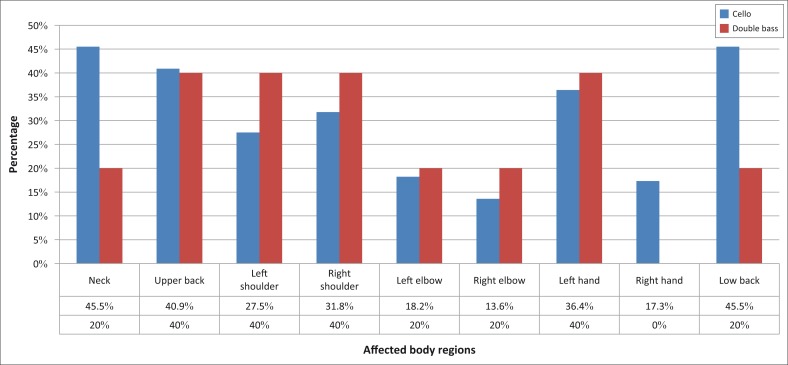
Distribution of musculoskeletal problems in the nine anatomic regions in lower string instrumentalists.

The distribution of PRMDs varied for the four bowed string instruments as illustrated in Figures 2 and 3. The upper string players (violinists and violists) reported problems mainly in the low back (81%) and the left shoulder (48%), whereas the lower string players (cellists and double bassists) reported problems mainly in the neck (46%) and the low back (46%).

**FIGURE 4:**
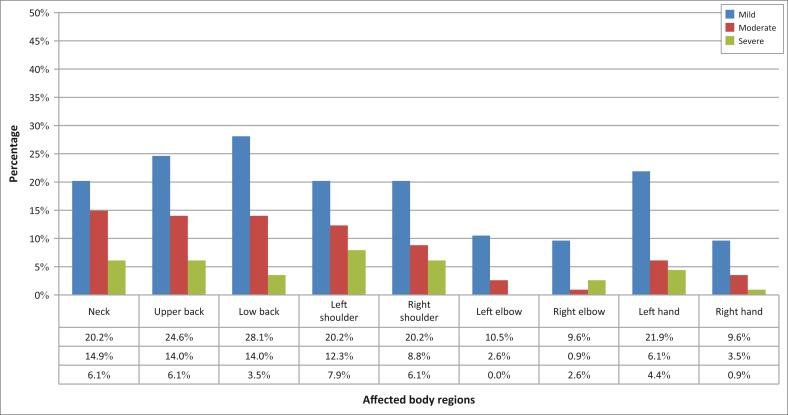
Illustrates the severity of the self-reported musculoskeletal problems across the nine anatomic body regions of the trunk and upper extremities.

When assessed for severity, self-reported problems were mild to moderate in the upper extremities and trunk. The left shoulder (8%), right shoulder (6%), upper back (6%) and neck (6%) were most commonly affected as illustrated by Figure 4.

### Association between risk factors and musculoskeletal problems

Gender was significantly associated with the presence of musculoskeletal problems especially in the trunk and left shoulder (*p* < 0.05). The unadjusted odds ratio using binary logistic regression showed that males were 0.31 times less likely to have musculoskeletal problems in one or more anatomic regions when compared to females (95% CI = 0.13–0.78). Table 3 shows the unadjusted and adjusted relationship using binary logistic regression between gender and the presence of musculoskeletal problems in the nine anatomic regions.

**TABLE 3:** Relationship between gender and the presence of musculoskeletal problems in the nine anatomic regions.

Anatomic regions	Unadjusted		Adjusted	
	OR (95% CI)	*p*	OR (95% CI)	*p*
Neck	0.16 (0.06–0.43)	0.00*	0.12 (0.04–0.37)	0.00*
Upper back	0.31 (0.13–0.72)	0.01**	0.38 (0.16–0.93)	0.03**
Low back	0.29 (0.12–0.69)	0.01**	0.29 (0.12–0.73)	0.01**
Right shoulder	0.46 (0.19–1.10)	0.08	0.42 (0.16–1.08)	0.07
Left shoulder	0.40 (0.71–0.94)	0.04**	0.41 (0.17–1.00)	0.05
Right elbow	0.46 (0.12–1.76)	0.26	0.46 (0.12–1.81)	0.27
Left elbow	0.71 (0.21–2.39)	0.57	0.44 (0.11–1.81)	0.26
Right hand	0.61 (0.22–1.70)	0.35	0.69 (0.24–1.98)	0.49
Left hand	0.66 (0.28–1.56)	0.34	0.53 (0.20–1.37)	0.19

* *p* < 0.01;

** *p* < 0.05.

Age (*U* = 963, *p* > 0.05), practice hours (*U* = 958, *p* > 0.05), instrument played (*p* > 0.05) and playing experience (*U* = 1055, *p* > 0.05) showed no significant association with the prevalence of musculoskeletal problems. However, age showed a significant association with the presence of musculoskeletal problems in the low back, left shoulder, right shoulder and left hand, whereas years of playing experience showed a significant association with problems in the low back, left shoulder and left hand (*p* < 0.05). There was no significant difference in the prevalence of musculoskeletal problems between the upper string (violin and viola) and the lower string (cello and double bass) groups (*p* > 0.05).

## Discussion

The results of this study showed that 77% of string instrumentalists reported problems in one or more anatomic regions with 35% currently experiencing problems that interfere with their performances. The prevalence of musculoskeletal problems in this study is similar to studies that reported a high prevalence of musculoskeletal problems among string instrumentalists (Abréu-Ramos & Micheo 2007; Leaver, Harris & Palmer 2011; Lederman 2003). The prevalence of problems reported is significantly higher among females when compared with males, especially in the trunk and the left shoulder. The increased prevalence of musculoskeletal injury among female string instrumentalists is similar to previous studies (Davies & Mangion 2002; Heming 2004). Risk factors such as joint laxity (Brandfonbrener 2002), hormonal differences (Anderson et al. 2001) and smaller body frame (Ekman et al. 2000) have been identified in literature as contributing factors to the increased prevalence of musculoskeletal problems reported in females than males. It has also been reported that females are more predisposed to reporting illnesses when compared to males (Barsky, Peekna & Borus 2001). These differences between males and females should be considered in developing and implementing an injury prevention programme.

The lower string players in this study reported a higher prevalence of problems when compared to the upper string players as also reported in earlier studies with a professional symphony orchestra (Abréu-Ramos & Micheo 2007) and young music students (Ranelli et al. 2011). Problems are reported in the neck, low back and left shoulder. This distribution is similar to studies performed on professional orchestra instrumentalists (Leaver et al. 2011; Paarup et al. 2012). Postural and bowing demands of playing the specific string instrument are consistent with the body regions affected, and this explains the variability in the distribution of musculoskeletal problems with regard to each string instrument (Paarup et al. 2012). The increased prevalence of musculoskeletal problems in the low back among violists when compared to violinists in this study is an interesting finding. This is especially because the violin and viola are similar in the postural position required for performance with the only difference being that the viola is a heavier instrument (Turner-Stokes & Reid 1999). The influence of the weight of instrument on the distribution of musculoskeletal problems might be important in proffering injury preventative programmes.

The severity of musculoskeletal problems in this study was reported as mild to moderate. A study conducted by Steinmetz et al. (2015) among professional orchestra musicians reported similar results using the numeric pain rating scale. The numeric pain rating score was between 3.9 and 4.7 in the affected body regions (Steinmetz et al. 2015). Although the complaints of pain in our study on string players were reported as mild to moderate in severity, it is worth noting that more players had severe pain in the upper back, neck and both shoulders compared to other affected regions as shown in Figure 4. Regardless of the mild–moderate severity of the complaints in this study, there is an increased severity of problems in the upper back, neck, right and left shoulder in comparison with the low back.

Prolonged neck flexion and the abnormal static posture of the left shoulder could be responsible for the increased severity in the upper trunk and left shoulder (Moraes & Antunes 2012), whereas repetitive bowing action could be responsible for the increased severity in the right shoulder. In playing the string instrument, the right shoulder involvement in repetitive bowing activity could result in musculoskeletal injury within the shoulder (Turner-Stokes & Reid 1999). Similar results indicate that postural imbalance is common among string instrumentalists, and postural structures are the most injured body regions in the string instrumentalist (Barczyk-Pawelec et al. 2012; Moraes & Antunes 2012). This implies that in-depth ergonomic assessment and nuanced corrective action could potentially reduce and prevent musculoskeletal problems among string players. Interventions up to now have involved a general rehabilitation approach and may not be addressing the specific areas needed in the intervention.

## Limitations of study

Self-administered questionnaire studies rely on the subjective opinion of the respondent and it can be biased (Bowling 2005; Cook 2010). The questionnaire was administered and completed by the orchestras at different music seasons of the year. Some completed the questionnaire at the peak of their season, whereas some completed the questionnaire off-season. The data collected might not represent the overall prevalence of musculoskeletal problems among string instrumentalists in South Africa. Because of the difficulty with responses, different modes (online and paper-based) of data collection were used and could have influenced the quality of the data (Bowling 2005). Musicians with a history of musculoskeletal problems are more likely to be motivated to respond to the survey when compared to those without history of musculoskeletal problems. This may therefore skew the reported prevalence.

## Conclusion

In conclusion, this study suggests that musculoskeletal problems of string instrumentalists are high in South Africa. The trunk, right and left shoulders are the most common areas of injury. An understanding of the most commonly affected body regions and the severity of injury coupled with the gender differences studied should be used in developing an injury prevention strategy to reduce the prevalence of musculoskeletal injury among string instrumentalists. In addition, an educational programme about causative factors and injury prevention strategies with the orchestra may be useful in developing a comprehensive injury prevention programme. Successful implementation of an effective programme is possible if health promotion is effected and collaboration is fostered between health practitioners and performers.
